# Quantitative microbial risk assessment of pathogen exposure from rainwater used in high-pressure vehicle washing

**DOI:** 10.2166/wh.2025.365

**Published:** 2025-03-01

**Authors:** John M. Johnston, Michael A. Jahne

**Affiliations:** a U.S. EPA Office of Research and Development, Center for Environmental Measurement and Modeling, Athens, GA 30605, USA; b U.S. EPA Office of Research and Development, Center for Environmental Solutions and Emergency Response, Cincinnati, OH 45268, USA

**Keywords:** disability-adjusted life year, microbial pathogens, non-potable, QMRA, rainwater

## Abstract

A literature-based quantitative microbial risk assessment (QMRA) was performed for the fit-for-purpose use of roof-collected rainwater in high-pressure vehicle washing. Our exposure assessment combined estimates of enteric pathogens in roof runoff (available for *Salmonella, Campylobacter*, and *Giardia* spp.) with an experimental study that directly measured vehicle washing exposure doses via a conserved tracer. For dose–response modeling, we considered a disability-adjusted life year (DALY) endpoint to capture the disease burden of potential pathogen infections. Annual risks for domestic and occupational scenarios were compared to a 10^−6^ DALY per person per year (ppy) benchmark using either untreated water or water treated to achieve previously reported log reduction targets (LRTs) for other forms of non-potable use. Combined across pathogens, vehicle washing using untreated roof-collected rainwater resulted in 95th percentile risks of 10^−1.4^ and 10^−2.4^ DALY ppy for occupational and recreational exposures, respectively, exceeding the selected benchmark. Treatment following indoor use or irrigation LRTs met the benchmark for domestic but not occupational use, suggesting that home vehicle washing can be included with other non-potable uses following existing treatment guidances. We also calculated new setting-specific LRTs for both scenarios (1.0–3.5 for domestic and 3.0–5.5 for occupational depending on pathogen), providing explicit risk-based treatment guidance for these applications.

## Graphical Abstract

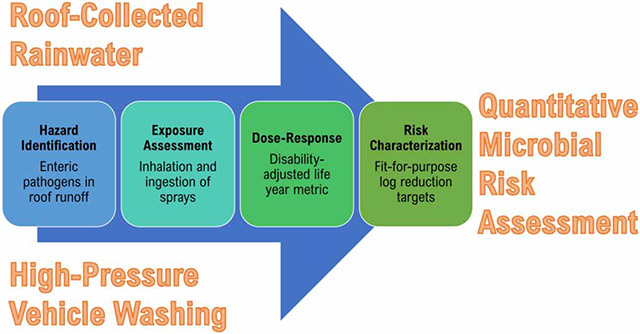

## INTRODUCTION

In this study, a literature-based quantitative microbial risk assessment (QMRA) was conducted to characterize potential enteric pathogen health risks from exposure to roof-collected rainwater used in high-pressure vehicle washing. A QMRA follows the general steps of hazard identification (What is driving the risk?), exposure assessment (What are the routes of exposure?), dose–response modeling (What is the effect of a given dose?), and ultimately risk characterization (What is the resulting risk estimate and is it acceptable?) (Haas et al. 2014). The QMRA provides a systems view of pathogen sources, fate and transport pathways in the environment, potential exposure routes, and relevant receptor populations (WHO 2003; U.S. EPA 2007; Ashbolt et al. 2010). The QMRA has been used to characterize human health risks in stormwater-impacted recreational waters (McBride et al. 2013; Soller et al. 2015), and it has been applied to planned use scenarios related to rainwater harvesting in urban environments (Ahmed et al. 2010a, b; Haas et al. 2014; Hamilton et al. 2017; Schoen et al. 2017; Hora et al. 2018; Clark et al. 2019; Denissen et al. 2021; Kusumawardhana et al. 2021; Pecson et al. 2022).

There are many competing demands for limited water supplies, including drinking water, agricultural, commercial, and industrial uses. In the face of climate change and a growing population, interest is increasing in the capture and use of precipitation collected onsite for landscape irrigation, cleaning, and other non-consumptive uses (Ghimire et al. 2014). However, the lack of risk-based standards for non-potable rainwater or stormwater use means that not all communities have adequate tools and guidance for public health decision-making regarding rainwater use.

‘Fit-for-purpose’ specifications of a reclaimed water application are designed to protect public health, the environment, or other needed endpoints based on the given source of water and its designated end use (U.S. EPA 2019). Such guidelines are informed by human health and ecological risk assessments designed to address specific exposure scenarios and risk management questions. This provides a risk-based alternative to the culturable fecal indicator bacteria (FIB) values contained in past water quality standards for collected rainwater quality (e.g., by U.S. states), which are often based on guidelines for recreational water.

Recreational water quality recommendations are designed to protect human health from incidental ingestion of ambient waters affected by fecal contamination and are informed by epidemiological studies characterizing waters with significant human fecal inputs (Prüss 1998; Wade et al. 2003, 2006). However, the mechanisms of fecal contamination of roof-collected rainwater are different than surface waters. Fecal contamination of surface waters can include human and non-human inputs from various point and non-point sources, whereas roof-collected rainwater quality can be affected by wildlife, such as birds and small mammals, and the deposition of microbes attached to airborne particulates (Ahmed et al. 2011; Schoen et al. 2017; Alja’fari et al. 2022). As a result, the relationships between FIB and enteric pathogens in roof-collected rainwater will be different compared to sewage-affected surface waters (Hörman et al. 2004; Ahmed et al. 2008, 2010a, b, 2011). FIB recommendations developed from studies characterizing sewage contamination in surface waters are, therefore, not appropriate for a risk-based evaluation of waters that are not sewage-impacted, such as roof-collected rainwater (Ashbolt et al. 2010). Exposure volumes may also differ greatly between recreational and designated non-potable uses, further limiting the applicability of FIB criteria tied to the former setting. While U.S. Recreational Water Quality Criteria (U.S. EPA 2012) are designed to achieve estimated illness rates of 32/1000 or 36/1000 illnesses per event, these metrics are specific to the underlying epidemiological studies and are not directly applicable to other water use scenarios.

Risk-based approaches address this challenge by considering the specific pathogen hazards in different sources of water (e.g., rainwater vs. surface water) and realistic exposure scenarios for the end use of interest in order to specify the level of pathogen reduction required to meet stated risk management goals (Petterson & Ashbolt 2016; Schoen et al. 2017). As reviewed by Jahne et al. (2023), fit-for-purpose enteric pathogen treatment targets have been developed for onsite non-potable reuse, including roof-collected rainwater (Schoen et al. 2017, 2023; Pecson et al. 2022). The seminal guidance document by Sharvelle et al. (2017), based on the work of Schoen et al. (2017), includes a limited range of end uses including toilet flushing, laundry, and landscape irrigation; it also includes a rare accidental ingestion to account for unintentional exposure or cross-connection to the non-potable water. Subsequent assessments proposed that indoor use treatment targets, which include the accidental ingestion factor, would also be protective for vehicle washing (Schoen et al. 2023), but a generalized exposure estimate was assumed and specific targets for this use were not developed in that work. Schoen et al. (2023) later updated the authors’ 2017 assessment to incorporate new information on pathogen characterizations and dose–response but did not expand the end uses considered. In their analysis, Pecson et al. (2022) extended the end uses to include vehicle washing based on coarse exposure assumptions from Australian guidelines for municipal irrigation (NRMMC 2006). However, a robust risk assessment specifically tailored to rainwater use in vehicle washing has not been conducted.

In the QMRA presented herein, annual risks for the fit-for-purpose use of reclaimed rainwater for vehicle washing are analyzed to assess risk-based enteric pathogen management needs. Reported pathogen characterizations in roof runoff (*Salmonella, Campylobacter*, and *Giardia* spp. based on available data) are combined with a tracer-based exposure model for high-pressure sprays to simulate potential risks using either untreated water or water treated to meet existing pathogen log reduction targets (LRTs) for other non-potable uses. New setting-specific LRTs are also developed for domestic and occupational scenarios to provide tailored treatment guidance for vehicle washing applications.

## METHODS

### QMRA model

This QMRA considered ingestion exposure to enteric pathogens during high-pressure vehicle washing with roof-collected rainwater. Annual risks were computed using a disability-adjusted life year (DALY) endpoint following the calculations of Schoen et al. (2023):

(1) $$\text{Dose}=10^{C}\times V\times 10^{-3}$$

(2) $${\text{DALY}}_{i}={\text{DR}}_{\text{inf}}[\text{Dose}]\times P_{\text{ill}|\text{inf}}\times {\text{DALY}}_{\text{ill}}$$

(3) $${\text{DALY}}_{\text{ann}}=1-\prod_{i=1}^{n}(1-{\text{DALY}}_{i})$$

where Dose is the ingestion dose per event (CFU or cyst equivalents), C is the source water concentration (log_10_CFU or cyst equivalents/L), V is the exposure volume (mL/event), ${\text{DALY}}_{i}$ is the disease burden per event (DALY/person/event), ${\text{DR}}_{\text{inf}}$ is the pathogen-specific dose–response function for the probability of infection, $P_{\text{ill}|\text{inf}}$ is the conditional probability of illness given infection (unitless), ${\text{DALY}}_{\text{ill}}$ is the disease burden per illness (DALY/illness), and ${\text{DALY}}_{\text{ann}}$ is the annual disease burden (DALY/person/year) for n exposures per year. Model inputs for pathogen distributions, dose–response models, and exposure assessment values are provided in Table 1 and described in subsections below.

We performed a Monte Carlo simulation (*n* = 10,000 iterations) to propagate the variability of input parameters to model output, resulting in a distribution of outcomes representing the potential range of risks. Annual DALYs per person per year (ppy) were compared to the World Health Organization benchmark of 10^−6^ DALY ppy for non-potable water reuse (WHO 2006). Sensitivity analysis using Spearman’s rank order correlation was used to assess the influence of each parameter on variability in risk estimates. The QMRA steps were conducted using R statistical software version 4.1.3 (R Core Team 2015), including the packages fitdistrplus version 1.1-8 (Delignette-Muller & Dutang 2015), rriskDistributions version 2.1.2 (https://cran.r-project.org/web/packages/rriskDistributions/), and mc2d version 0.1-21 (Pouillot & Delignette-Muller 2010).

### Reference pathogens and dose–response

Reference pathogens were selected based on those detected in the U.S. roof runoff study by Alja’fari et al. (2022) and included *Salmonella* spp., *Campylobacter* spp., and *Giardia lamblia/duodenalis/intestinalis* species complex (‘*Giardia*’ herein; see ‘Pathogen characterization‘ below). Dose–response modeling was performed using current dose–response relationships from the scientific literature as recommended by Schoen et al. (2023) (Table 1). *Salmonella* spp. and *Giardia* utilized approximate beta-Poisson and exponential functions for the probability of infection, respectively, with dose-independent conditional probabilities of illness: *Campylobacter* spp. used a hypergeometric function (i.e., exact beta-Poisson) for the probability of infection and a dose-dependent conditional probability of illness. DALY per illness values were adopted from Schoen et al. (2023) based on a North American study of foodborne illness (Havelaar et al. 2015); refer to Schoen et al. (2023) for discussion.

### Pathogen characterization

For rainwater source characterization, we utilized the distributions reported by Schoen et al. (2023) for consistency in resulting risk guidances. For bacterial distributions (*Campylobacter* and *Salmonella* spp.), the fecal contamination of roof runoff (g feces/L) was estimated based on the relative FIB densities in roof animal feces and freshly collected roof runoff samples. This was then coupled with reported pathogen densities in the animal feces (CFU/g feces) to model resulting pathogen concentrations in roof runoff (CFU/L) (Schoen et al. 2017). As noted by Schoen et al. (2017), sufficient data were only available to model one animal type (gulls) and no data were available for relevant zoonotic protozoa (*Giardia* and *Cryptosporidium* spp.). Pecson et al. (2022) instead utilized the empirical pathogen measurements by Alja’fari et al. (2022), which collected approximately 80 samples from four U.S. cities over 13 storm events. Despite its 10 L sample volume, however, the study experienced high levels of non-detects (91.1, 97.5, and 94.9% for *Salmonella* spp., *Campylobacter* spp., and *Giardia*, respectively), limiting data interpretability for QMRA. Given this uncertainty, Pecson et al. (2022) used both lognormal (fitted to the observed data with non-detects set to the detection limit) and uniform distributions (observed range adjusted by detection rate) without preference between them. Schoen et al. (2023) selected their uniform approach for *Giardia* to address the modeling data gap, assuming 100% occurrence consistent with the other pathogens that were detected in nearly all composited fecal samples (Schoen et al. 2017). A first flush diversion was not considered by either the modeling or empirical characterization approach.

### Exposure assessment

Sinclair et al. (2016) quantified incidental water ingestion during 10 min of car wash activity with a high-pressure spray device. They used cyanuric acid (CYA), a pool chemical used to stabilize chlorine, as a conserved tracer that provides an accurate measure of actual exposure (Dufour et al. 2006). Urine was collected from research participants over a 24-hour period post-exposure. By measuring the amount of CYA recovered, the authors were able to back-calculate the combined volume of ingested water and inhaled aerosols during the car wash activity. Seventy percent of participants had measurable ingestion volumes ranging from 0.06 to 3.79 mL (Sinclair et al. 2016). The authors used Monte Carlo simulation to generate a spray ingestion volume distribution from their study data, accounting for the variability in excretion between individuals with a mean of 0.438 mL (standard deviation 1.24), a geometric mean of 0.090 mL, and 95th percentile of 1.93 mL (Sinclair et al. 2016). In their later publication (Barker et al. 2017), the authors fitted this data to a lognormal distribution, which is adopted here (Table 1). Annual risk was calculated using domestic and occupational exposure scenarios proposed by Sinclair et al. (2016), with the modification that domestic exposure included car washing only (i.e., not window or hard surface washing) (Table 1). Following Sinclair et al. (2016), volumes for events with a longer duration than the 10-min exposure study were calculated based on the number of 10-min increments included (e.g., 6 h ¼ 36 × 10 min).

### Log reduction targets

If the risk benchmark was not met using untreated water, previously developed LRTs for other forms of non-potable roof runoff reuse (Table 2) were applied to consider whether they would also be protective for this application. The LRTs are from Schoen et al. (2023) and Pecson et al. (2022), who used the aforementioned modeling and empirical dataset approaches, respectively, to characterize pathogen distributions (previous section). In both cases, indoor use comprises toilet flushing and clothes washing with a rare accidental ingestion event, and irrigation comprises unrestricted-access landscape or municipal irrigation and dust suppression (Pecson et al. 2022; Schoen et al. 2023). Schoen et al. (2023) reported LRTs for both the 10^−6^ DALY ppy benchmark and one of 10^−4^ infections ppy; Pecson et al. (2022) reported the latter only. Note that Schoen et al. (2023) updated the authors’ previous analysis (Schoen et al. 2017) to incorporate updated dose–response information for *Campylobacter* spp., superseding those results; *Salmonella* spp. LRTs remained the same.

The impact of implementing the previous LRTs on resulting risk was assessed by subtracting the log_10_ reduction value (LRV) associated with each DALY-based LRT from Schoen et al. (2023) (i.e., for indoor use and irrigation) from the pathogen concentration C (log_10_CFU or cyst equivalents/L) in Equation (1) to represent the concentration following that level of treatment; the DALY set was selected for consistency with the risk metric used here. New setting-specific LRTs for domestic and occupational use were also calculated by iteratively modifying C to subtract hypothetical LRVs in 0.5 log_10_ increments, selecting the value that achieved the selected risk benchmark in 95% of simulations.

## RESULTS

**Figure 1 |:**
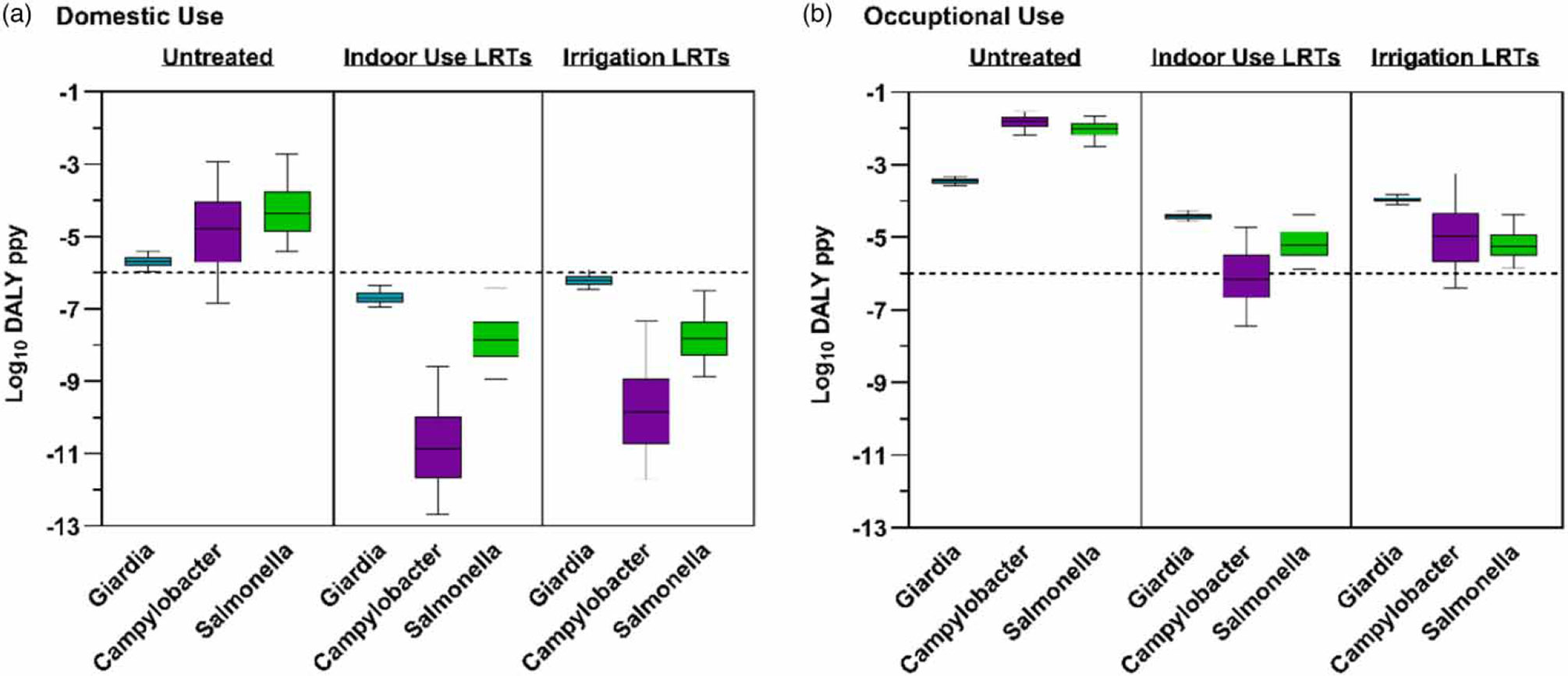
Estimated disease burden in DALY ppy for simulated exposure to enteric pathogens in roof-collected rainwater used for high-pressure vehicle washing in (a) domestic and (b) occupational settings. Risks for untreated water and water treated to meet the Schoen et al. (2023) LRTs for indoor non-potable use or irrigation following a 10^−6^ DALY ppy benchmark are compared to that metric (dashed lines). Boxplots represent the median and upper/lower quantiles and whiskers indicate the 95th percentile range.

High-pressure vehicle washing using roof-collected rainwater resulted in predicted risks that exceed the 10^−6^ DALY ppy benchmark (Figure 1). The highest modeled disease burdens were for the bacterial pathogens *Campylobacter* spp. And *Salmonella* spp. followed by *Giardia* and greater for occupational vs. domestic scenarios. Combined risks across pathogens were 10^−1.4^ and 10^−2.4^ DALY ppy for occupational and domestic exposures, respectively. Treatment following the Schoen et al. (2023) DALY-based LRTs for indoor non-potable use or irrigation met the 10^−6^ DALY ppy benchmark for the domestic scenario (95th percentile *Giardia* risk following the irrigation LRT was 10^−5.9^ DALY ppy and assumed to be within the margin of model error). Comparison of setting-specific LRTs for domestic use (Table 3) to the other previous LRT sets (Table 2) indicates that they would also be protective for domestic vehicle washing applications. In contrast, occupational use exceeded the DALY benchmark following treatment to the previous DALY-based LRTs and new LRTs for this scenario are proposed (Table 3); the other prior sets (Table 2) would also not achieve these for all pathogens. Simulated risks for the bacterial reference pathogens were more sensitive to modeled concentrations than exposure volume (*Campylobacter* spp. ρ=0.93vs.ρ=0.34, respectively; *Salmonella* spp. ρ=0.97vs.ρ=0.21). *Giardia* concentrations were modeled using a bounded uniform distribution resulting in more similar sensitivity to either variable (ρ=0.69vs.ρ=0.67 for volume).

## DISCUSSION

The QMRA has been used to evaluate the human health risks associated with rainwater use, including the potential microbial risks to households consuming their homegrown produce (Lim & Jiang 2013). This evaluation includes exposure to the opportunistic pathogens such as *Legionella pneumophila* and *Mycobacterium avium* from activities such as showering, swimming, using a garden hose, washing cars, and flushing toilets (Hamilton et al. 2017). It also considers the infection risk asociated with *L. pneumophila* from toilet flushing (Kusumawardhana et al. 2021), as well as exposure to human pathogenic bacteria like *M. tuberculosis, Yersinia* spp., and *Listeria monocytogenes* from home cleaning, laundry washing, and garden hose use (Denissen et al. 2021). Additionally, the evaluation examines the risk of *Campylobacter* spp. infections from bathing, food washing, garden hose use, and toilet flushing (Hora et al. 2018). It includes the influence of roof material on pathogen exposure from consuming lettuce irrigated with roof-collected rainwater (Clark et al. 2019); and the infection risk of *Salmonella, G. lamblia, L. pneumophila*, and *Campylobacter jejuni* from exposure during showering, drinking, and garden hose use (Ahmed et al. 2010a, b). In two of these studies, it was determined that annual pathogen infection risk was greater than 10^−4^ ppy, with the recommendation to treat roof-collected rainwater for non-potable uses (Lim & Jiang 2013; Kusumawardhana et al. 2021).

Based on bacterial and protozoan pathogen annual DALY risks in the current study, the management of pathogen exposure is needed for regular domestic or occupational use of rainwater in high-pressure vehicle washing. Previous risk-based treatment guidances have specified pathogen LRTs for onsite non-potable use of roof runoff, likewise identifying a need to reduce pathogen exposure (Schoen et al. 2017, 2023; Sharvelle et al. 2017; Pecson et al. 2022). Although vehicle washing was not included in the selection of non-potable end uses considered by Schoen et al. (2017) and Sharvelle et al. (2017) nor the updated analysis by Schoen et al. (2023), previous work demonstrated that the determined LRTs are also protective for this application (Schoen et al. 2023). The LRTs developed by Pecson et al. (2022) explicitly considered vehicle washing as an end use and, in contrast to Sharvelle et al. (2017), included protozoan targets based on *Giardia* measurements reported in Alja’fari et al. (2022) (as did Schoen et al. (2023)). We built on this work by using an activity-specific exposure model based on conservative tracer measurements (Sinclair et al. 2016), rather than assuming comparable exposure to monthly municipal irrigation (NRMMC 2006). Our results demonstrate that the previous LRTs for indoor non-potable use and irrigation are also protective for enteric pathogen management during high-pressure vehicle washing with roof-collected rainwater in domestic settings (10 min weekly). However, given the longer exposure duration and higher frequency of occupational use (6 h daily for 240 days), treatment to meet the prior LRTs did not achieve the 10^−6^ DALY ppy benchmark in this setting. New LRTs are presented in this work that may be used to develop vehicle washing-specific rainwater treatment policies.

Limitations of this study include the reliance on literature data for rainwater pathogen concentrations, which were an influential factor in risk model predictions. Our risk assessment follows the concept of reference pathogens, focusing on the enteric pathogens likely to drive infection risks and for which necessary data are available; it is assumed that LRTs derived for this subset are also protective of other pathogens within their classes (e.g., pathogenic *Escherichia coli* for enteric bacteria) (Schoen et al. 2017). However, these reference pathogen selections are not exhaustive and do not represent all potentially human-infectious organisms in rainwater, excluding alternative fecal-borne zoonotic pathogens not considered or detected by existing characterization studies. For example, although Alja’fari et al. (2022) did not detect *Cryptosporidium* spp. in any samples, this does not preclude its potential occurrence in rainwater, as has been observed in other international studies (Ahmed et al. 2010a, b). This underscores the potential benefits of additional pathogen data collection to ensure that the full variability of their concentrations has been accurately characterized. However, it should be noted that Alja’fari et al. (2022) attempted to perform a representative, national characterization of rainwater pathogens to serve as input to national and regional QMRAs, and their dataset demonstrated the challenges of characterizing rainwater with a high number of non-detects as well as regional differences in pathogen presence and concentrations. Improved data on pathogen densities in the feces of different roof-access animals would therefore also be beneficial when applying the modeling approach of Schoen et al. (2017); currently, it is limited to one type of bird (gulls). Since opportunistic pathogens such as *Legionella* and *Mycobacterium* spp. grow within premise plumbing environments and are therefore best controlled using distribution system best management practices, we did not assess their need for exposure reduction from source water; associated recommendations for onsite non-potable water systems are available elsewhere (Sharvelle et al. 2017).

Sensitivity analysis indicated that the ingestion rate also contributed to variability in risk estimates. This emphasizes the importance of our improved exposure assessment, which uses the ingested/inhaled volume distribution from Sinclair et al. (2016), in contrast to previous assessments that relied on simplified exposure estimates adopted from the Australian recycled water guidelines for different end uses (i.e., municipal irrigation) (NRMMC 2006; Pecson et al. 2022). Schoen et al. (2017) estimated that the municipal irrigation volume (10^−3^ L) would equal approximately one drop of water or 10–100 seconds of wet hand-to-mouth contact. In contrast, Sinclair et al. (2016) directly assessed internal-effective exposure volume in their experiment, developing an adjusted volume distribution that accounted for individual variation using Monte Carlo analysis. Using this method, no assumptions regarding routes of exposure, proportion of inhaled versus ingested fractions, or the effect of particle size distribution on aerosol deposition were required.

Our study utilizes a new development in dose–response modeling for QMRA practice. Recent work has reevaluated the dose–response data for *C. jejuni* using a multilevel model that accounted for data from both challenge studies and outbreaks (Teunis et al. 2018). Interestingly, the analysis noted a high rate of infection among subjects with a lower likelihood of acute gastrointestinal symptoms dependent on the exposure dose. This dose-dependent probability of illness contrasts with dose–response models for the other reference pathogens in our study, which assume a constant conditional probability of illness based on earlier work. Schoen et al. (2023) also highlight this pattern and its influence on risk-based decision-making when DALY metrics are used, further suggesting that additional assessment to determine whether other pathogens also follow dose-dependent probability of illness relationships is warranted.

This study adds to the body of work, suggesting that pathogen exposure control may be necessary during the risk-based management of rainwater collection and use. Previous studies have recommended LRTs ranging from 1.0 to 3.5 for enteric bacteria and 1.0–1.5 for parasitic protozoa (Jahne et al. 2023). These LRTs were shown by the current analysis to also be protective for domestic vehicle washing scenarios. To meet the targets, Pecson et al. (2022) provide a model treatment train utilizing cartridge filtration for pathogen removal followed by secondary disinfection with chlorine to reduce fouling and biofilm production in plumbing and storage systems, including the potential for opportunistic pathogen growth (e.g., *Legionella*). Alternative management strategies to reduce pathogen exposure could also be used, such as first flush diversion, i.e., discarding the initial collection of roof runoff that is most likely to contain previously deposited contaminants. Because the rainwater characterization methods did not include this process, which is common in rainwater collection practice, the risk assessment herein provides a conservative estimation of pathogen load and therefore risk. However, previous studies suggest that first flush diversion alone is insufficient to prevent the bacterial contamination of rainwater collections (Gikas & Tsihrintzis 2012) and future work is needed to quantitatively characterize its impact on pathogen concentrations and associated risk modeling outcomes. Personal protective equipment (PPE) such as respirators can also be used to control exposure, particularly in occupational settings where such practices are commonly established. Notably, PPE use would also reduce potential exposure to *Legionella*, which, although not considered in the current enteric pathogen risk assessment, has been noted by others as a reuse concern (Hamilton et al. 2017; Sharvelle et al. 2017). Our results indicate that occupational vehicle washing, which occurs more frequently and for longer durations, requires approximately 2 log_10_ greater pathogen exposure reduction than domestic uses; this may be achieved through additional treatment (e.g., ultraviolet disinfection) or supplementary management strategies such as PPE.

## CONCLUSIONS

Our study presents a novel QMRA of incidental ingestion during high-pressure vehicle washing with roof-harvested rainwater to inform decision-making regarding potential needs for pathogen exposure reduction. While future research should aim to improve the microbial characterization of roof runoff using measurement and or/modeling approaches, we utilize the best available information to develop specific risk-based guidance for this application to complement the existing guidance for other forms of onsite non-potable water reuse. The conclusions and pathogen reduction targets herein provide practical information to guide risk management policy for both domestic and occupational settings.

## Figures and Tables

**Table 1 |:** Inputs for roof-collected rainwater high-pressure vehicle washing QMRA

	Variable	Model	Units	Parameters	Values	References
**Pathogen characterization**	*Salmonella* spp. concentration	Empirical^a^	log_10_CFU/L	Median, 95th percentile	0.15	Schoen et al. (2017, 2023)
					3.94	
	*Campylobacter* spp. concentration	Empirical^a^	log_10_CFU/L	Median, 95th percentile	−0.85	Schoen et al. (2017, 2023)
					1.67	
	*Giardia* concentration	Uniform	log_10_cysts/L	Minimum	−0.7	Alja’fari et al. (2022), Pecson et al. (2022), Schoen et al. (2023)
				Maximum	1.2	
**Exposure assessment**	10-min exposure volume	Lognormal	mL	Mean^b^	0.45	Barker et al. (2017)
				Standard deviation^b^	0.88	
	Domestic exposure duration	Point estimate	minutes	Point estimate	10	Sinclair et al. (2016)
	Occupational exposure duration	Point estimate	minutes	Point estimate	360	Sinclair et al. (2016)
	Domestic exposure frequency	Point estimate	events/year	Point estimate	52	Sinclair et al. (2016)
	Occupational exposure frequency	Point estimate	events/year	Point estimate	240	Sinclair et al. (2016)
**Dose-response**	*Salmonella enterica* infection	Approximate beta-Poisson	Unitless probability	α	0.3126	Fazil (1996)
				β	2,884	
	*S. enterica* illness^c^	Uniform	Unitless probability	Minimum	0.17	Teunis et al. (1999)
				Maximum	0.4	
	*S. enterica DALY*	PERT	DALY/illness	Minimum	0.02	Havelaar et al. (2015), Schoen et al. (2023)
				Mode	0.03	
				Maximum	0.05	
	*C. jejuni* infection	Hypergeometric	Unitless probability	α	0.44	Teunis et al. (2018)
				β	0.51	
	*C. jejuni* illness^c,d^	Bivariate normal	Unitless probability	Mean (w)	−2.744	Teunis et al. (2018)
				Mean (z)	−0.0049	
				Variance (w)	1.337	
				Covariance (w, z) Variance (z)	0.01 0.993	
	*C. jejuni DALY*	PERT	DALY/illness	Minimum	0.02	Havelaar et al. (2015), Schoen et al. (2023)
				Mode	0.03	
				Maximum	0.05	
	*G. lamblia* infection	Exponential	cysts	r	0.0199	Rose et al. (1991)
	*G. lamblia* illness^c^	Uniform	cysts	Minimum	0.2	Eisenberg et al. (1996)
				Maximum	0.7	
	*G. lamblia DALY*	Point estimate	DALY/illness	Point estimate	0.003	Havelaar et al. (2015), Schoen et al. (2023)

CFU, colony forming unit; DALY, disability-adjusted life year.

a Refer to Schoen et al. (2017) for the calculation of the empirical distribution.

b Sample mean and standard deviation; population parameters were calculated following Barker et al. (2017).

c Conditional probability of illness given infection.

d Dose-dependent; formulas are provided in Teunis et al. (2018).

**Table 2 |:** Previously reported pathogen LRTs for onsite non-potable roof runoff use

End use	References	*Giardia*	*Campylobacter*	*Salmonella*
**Irrigation**	Schoen et al. (2023) ^a^	0.5	2.5	3.5
	Schoen et al. (2023) ^b^	1.5	5.0	3.5
	Pecson et al. (2022) ^b,c^	1.0	–	–
**Indoor use**	Schoen et al. (2023) ^a^	1.0	3.0	3.5
	Schoen et al. (2023) ^b^	2.0	5.0	3.5
	Pecson et al. (2022) ^b,c^	1.5	–	–

*Note.* Values are rounded to 0.5 log_10_ units.

a Benchmark of 10^−6^ DALY ppy.

b Benchmark of 10^−4^ infections ppy.

c Bacteria LRTs were calculated but not recommended, assuming that treatment for *Giardia* will also manage bacterial pathogens.

**Table 3 |:** Setting-specific log_10_ reduction targets for the use of roof-collected rainwater in high-pressure vehicle washing following a 10^−6^ DALY ppybenchmark

Setting	*Giardia*	*Campylobacter*	*Salmonella*
**Domestic**	1.0	2.0	3.5
**Occupational**	3.0	4.5	5.5

## Data Availability

All relevant data are available from an online repository or repositories: https://data.gov/.

## HIGHLIGHTS

- Our literature-based QMRA combined pathogen estimates from roof runoff with an experimental study of vehicle washing ingestion exposure.
- High-pressure vehicle washing with untreated rainwater resulted in risks that exceeded a 10^−6^ DALY ppy benchmark.
- Log reduction targets (LRTs) for other non-potable uses are protective for domestic but not occupational scenarios.
- New fit-for-purpose LRTs provide specific risk-based guidance.
